# The senescence-inhibitory p53 isoform Δ133p53α: enhancing cancer immunotherapy and exploring novel therapeutic approaches for senescence-associated diseases

**DOI:** 10.1007/s11357-025-01819-y

**Published:** 2025-08-06

**Authors:** Shinji Nakamichi, Leo Yamada, Christopher Roselle, Izumi Horikawa, Carl H. June, Curtis C. Harris

**Affiliations:** 1 Laboratory of Human Carcinogenesis, Center for Cancer Research, National Cancer Institute, National Institutes of Health, Bethesda, MD 20892 USA; 2 Center for Cellular Immunotherapies, Perelman School of Medicine, University of Pennsylvania, Philadelphia, PA 19104 USA; 3 Present Address: Boston Consulting Group, 1735 Market Street, Philadelphia, PA 19103 USA

**Keywords:** Δ133p53α, Cellular senescence, Chimeric antigen receptor T cells, Progeria, Pulmonary fibrosis, Transgenic mice

## Abstract

Δ133p53α is a naturally occurring isoform of the tumor suppressor protein p53. Δ133p53α functions as a physiological dominant-negative inhibitor of the full-length p53 protein (commonly referred to as p53). Δ133p53α preferentially inhibits p53-mediated cellular senescence, while it does not inhibit, or may even promote, p53-mediated DNA repair. Owing to this selective inhibitory activity that preserves genome stability, Δ133p53α represents a promising target for enhancement in the prevention and treatment of diseases associated with increased senescence of normal cells. These diseases include Alzheimer’s and other neurodegenerative diseases, premature aging diseases such as Hutchinson-Gilford progeria syndrome (HGPS), and idiopathic pulmonary fibrosis (IPF). Current cell-based therapies, which are limited by increased cellular senescence, may also benefit from Δ133p53α-mediated improvements. As an initial application of Δ133p53α in improving therapeutic cells, we here introduce Δ133p53α-armored chimeric antigen receptor (CAR)-T cells. Based on our previous and ongoing studies using various types of senescent human cells in vitro, we also discuss the importance of further exploring the therapeutic potentials of Δ133p53α, with particular focus on HGPS and IPF. The development of mouse models facilitates in vivo evaluation of the therapeutic effects of Δ133p53α, potentially leading to future clinical applications.

**Graphical Abstract:**

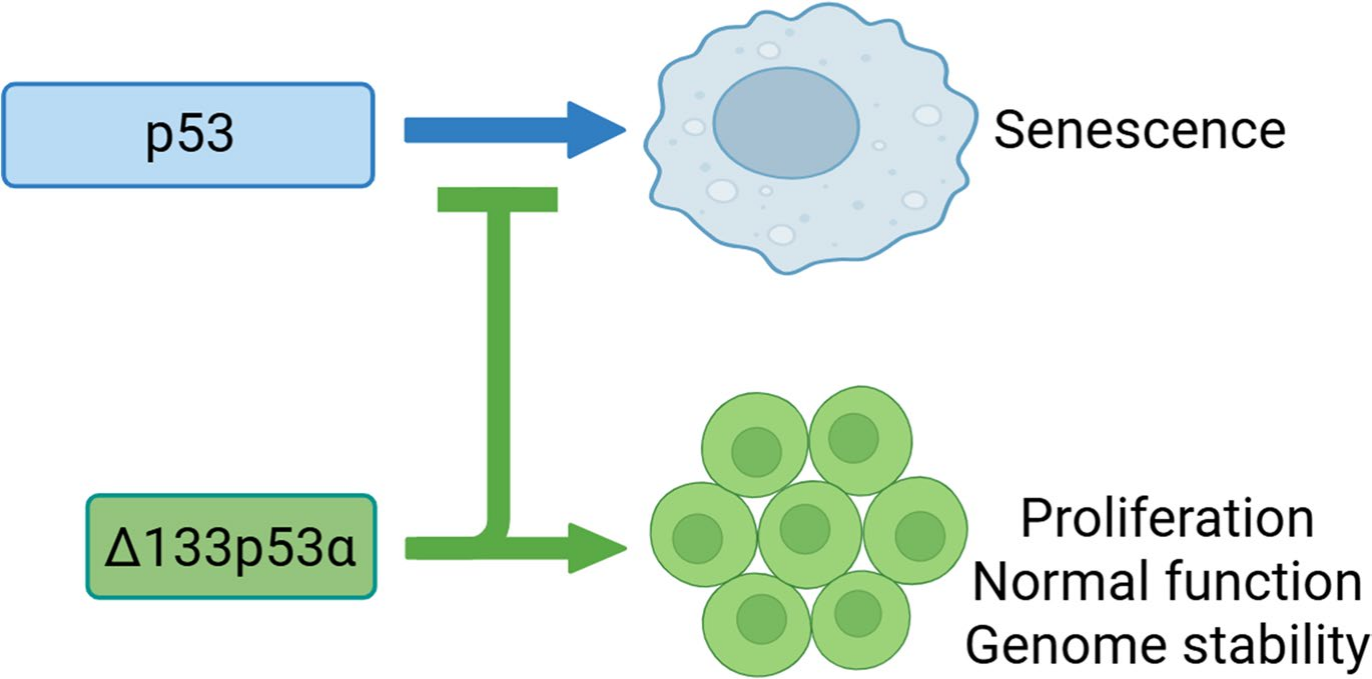

## Cellular senescence in aging and diseases

Cellular senescence, an irreversible or sustained arrest in cell proliferation, can function as a tumor suppression mechanism when induced in pre-cancerous or cancerous cells [1]. On the other hand, cellular senescence occurring in normal cells may contribute to organ dysfunction and aging phenotypes [1–3]. Cellular senescence occurs during mammalian embryonic development and plays a physiological role in proper tissue patterning [4, 5]. It is also critical for wound healing and tissue regeneration [3, 6, 7]. These beneficial and adverse effects of cellular senescence are mediated at least in part by the senescence-associated secretory phenotype (SASP). SASP involves various types of secreted molecules such as cytokines, chemokines, growth factors, proteases, and extracellular vesicles, which may be differentially induced by diverse types and levels of cellular stresses, including telomeric or non-telomeric DNA damage, reactive oxygen species (ROS), oncogene activation, and metabolic imbalance [8–10]. This article focuses on cellular senescence and proinflammatory SASP in normal cell types, which contribute to senescence-associated diseases and adverse conditions, as well as a p53 isoform-based approach for the potential prevention and treatment of these diseases and conditions.

## Δ133p53α: a natural p53 isoform that dominant-negatively inhibits p53-mediated senescence

The human *TP53* gene encodes not only full-length p53 protein (simply referred to as p53 in this article) but also at least a dozen naturally occurring protein isoforms due to alternative RNA splicing, alternative transcription initiation, and/or alternative translation initiation [11]. Among them, Δ133p53α is an N-terminally truncated isoform that is transcribed from the alternative promoter within intron 4 and translated from a methionine codon corresponding to amino acid residue 133 [11, 12]. Since this methionine codon is not evolutionarily conserved and is present only in human and primates [13], no equivalent protein isoform is expressed endogenously in non-primate organisms. In contrast to p53 subject to proteasomal degradation, Δ133p53α is degraded via chaperone-assisted selective autophagy in normal human cells undergoing senescence [13–15]. While downregulation of endogenous Δ133p53α is commonly observed in various types of senescent human cells (examples mentioned below) [13, 15–20], human pluripotent stem cells such as embryonic stem cells and induced pluripotent stem cells (iPSC) consistently express high levels of endogenous Δ133p53α [21]. The exogenous expression of Δ133p53α enhanced the efficiency of iPSC generation induced by Yamanaka factors from normal human fibroblasts [21], further supporting the functional link of Δ133p53α to cellular stemness [22]. Δ133p53α preferentially inhibits the p53-inducible genes contributing to cellular senescence, such as p21^WAF1^ and miR-34a [21], which are thus increased in Δ133p53α-downregulated senescent cells [16, 19, 20] and repressed in Δ133p53α-upregulated pluripotent stem cells [21]. Δ133p53α physically interacts with p53 and dissociates it from the promoter regions of the *p21*^*WAF1*^ and *miR-34a* genes [21], providing a mechanistic basis for its dominant-negative inhibition of p53. Notably, Δ133p53α does not impair p53-mediated maintenance of genome stability [21, 22] or may even promote DNA repair [19, 20]. These characteristics of Δ133p53α inhibiting cellular senescence and preserving genome stability are in marked contrast to complete inhibition of p53 activities, which could lead to genome instability and increased tumorigenesis [23, 24]. These background findings have prompted us to consider potential applications of Δ133p53α in prevention and treatment of senescence-associated diseases, as well as in improvement of therapies currently limited by cellular senescence.

## Investigation of Δ133p53α in CD8^+^ T cells and development of Δ133p53α-armored CAR-T cells

Following our initial study on Δ133p53α using normal human fibroblasts [16], we sought a cell type that undergoes cellular senescence in vivo and is relevant to human health and disease treatment. CD8^+^ T cells were selected, as they can be readily isolated from blood and separated into senescent and non-senescent cell populations using cell surface markers (e.g., the costimulatory receptor CD28-negative and the carbohydrate epitope CD57-positive in senescent populations) [25–27]. CD8^+^ T cells with a CD28^+^/CD57^−^ senescent phenotype significantly increase in number with donor age [15]. Compared to non-senescent populations, these cells show increased senescence-associated β-galactosidase (SA-β-gal) staining, a widely used senescence marker, accumulated DNA damage, elevated production of the proinflammatory SASP cytokines IL-6 and IL-8, and limited replicative capacity when transferred to in vitro cell culture [15]. The endogenous expression of Δ133p53α in these senescent CD8^+^ T cells was significantly lower than in non-senescent populations [15]. While CD28^−^ CD8^+^ cells underwent only ~ 2 population doublings in vitro, lentiviral vector-driven expression of Δ133p53α delayed proliferation arrest and extended the replicative lifespan by additional ~ 4 population doublings [15]. In these Δ133p53α-expressing, lifespan-extended CD8^+^ T cells, IL-6 and IL-8 production was reduced, CD28 expression was restored in 20% of the cells, and T cell stemness-associated proteins CD27 and CD62L were upregulated [15]. Moreover, inhibitory immune checkpoint receptors PD-1 and LAG-3 were downregulated upon Δ133p53α expression [15]. These data suggest that CD8^+^ T cell senescence contributes to the functional decline in T cell immunity in the elderly, and that Δ133p53α prevents CD8^+^ T cells from entering senescence and reprograms them to a stem-like, less differentiated and more potent state. The features induced by Δ133p53α are characteristic of T cells that are preserved against aging-associated changes and exhibit enhanced efficacy in cancer immunotherapy [28–32].

**Fig. 1:**
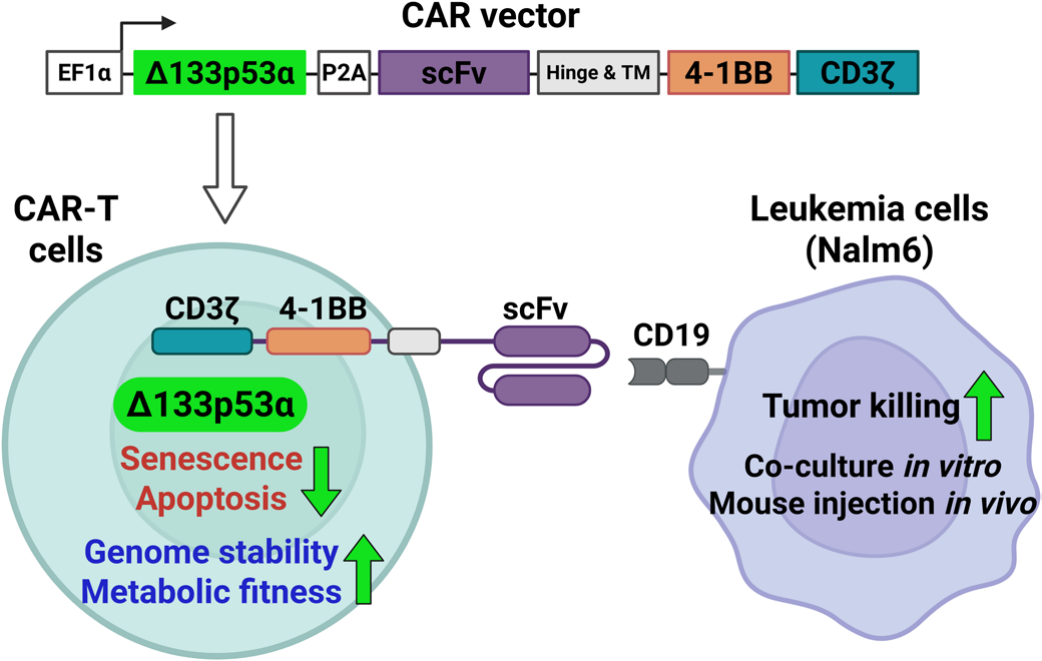
Superior anti-tumor activity of Δ133p53α-armored CAR-T cells. The expression cassette of Δ133p53α with a P2A self-cleaving peptide was inserted into the EF1α promoter-driven CAR vector, which encodes a scFv fragment recognizing CD19, the hinge and transmembrane domain, the 4-1BB costimulatory domain, and the CD3ζ activation domain. In the control vector, mCherry2 was inserted instead of Δ133p53α. The CAR-T cells transduced with these vectors were examined in tumor killing assays with a CD19-positive Nalm6 leukemia model. The Δ133p53α-armored CAR-T cells, compared to the mCherry2 control, exerted superior anti-tumor activity both in co-culture experiments in vitro and mouse injection experiments in vivo. Mechanistically, the gene expression profiling and cell metabolic assays suggested that the Δ133p53α-armored CAR-T cells were associated with decreased cellular senescence and apoptosis, as well as enhanced genome stability and metabolic fitness. The original data were published in Roselle C. et al. [34]. Created in BioRender.com

To test the hypothesis that Δ133p53α may improve cancer immunotherapy, we have integrated the Δ133p53α sequence into a clinically used CD19-directed CAR vector [33, 34] (Fig. 1). This Δ133p53α-armored CAR vector, in parallel to control vector, was transduced to T cells derived from normal donors or patients with chronic lymphocytic leukemia (CLL). The resulting CAR-T cells were evaluated for anti-tumor activity in a widely used CD19-positive leukemia model (Nalm6). In CAR-T/Nalm6 co-culture experiments, both Δ133p53α-armored and control CAR-T cells similarly resulted in complete tumor cell killing under conditions of low tumor burden (i.e., CAR-T: Nalm6 ratio = 1:2.5 or 1:5). In contrast, Δ133p53α-armored CAR-T cells showed significantly greater anti-tumor activity than control CAR-T cells under high tumor burden (i.e., CAR-T: Nalm6 ratio = 1:10 or 1:12.5) [34]. In a xenograft mouse model in which Nalm6 and CAR-T cells were injected intravenously, Δ133p53α-armored CAR-T cells exhibited superior tumor clearance in vivo, resulting in improved survival in mice injected with these CAR-T cells compared to those injected with the control cells [34]. These results indicate that Δ133p53α may confer enhanced and prolonged anti-tumor activity on CAR-T cells under nutrient-competitive conditions, such as in the presence of an overwhelming number of tumor cells in vitro or within the tumor microenvironment in vivo. Consistently, Δ133p53α-armored CAR-T cells exhibited enhanced metabolic fitness reflecting a hybrid metabolic state exemplified by both superior glycolysis and oxidative phosphorylation [35, 36]. Our gene expression profiling suggested that Δ133p53α functions to inhibit p53-mediated senescence and enhance genome stability in CAR-T cells [34], as previously observed in other cell types in our studies [19–21]. The p53-inducible genes for apoptotic cell death were also downregulated in Δ133p53α-armored CAR-T cells [34], likely contributing to their prolonged survival. The Δ133p53α-mediated inhibition of senescence and apoptosis, as well as enhancement of genome stability and metabolic fitness, well explains the Δ133p53α-mediated functional improvement of CAR-T cells (Fig. 1). Of clinical importance, Δ133p53α was able to functionally improve CAR-T cells derived from CLL patients, who failed to respond to standard CAR-T cell therapy currently in clinical use [34], supporting a potential application to non-responsive and refractory cases. Toward treatment of currently hard-to-treat types of solid tumors, modifications of the Δ133p53α-armored CAR vector and combinations with other improving strategies are to be considered.

## Astrocyte senescence, SASP, and neurotoxicity mitigated by Δ133p53α

A series of our studies using normal human astrocytes in cell culture and brain tissues from patients with Alzheimer’s disease, amyotrophic lateral sclerosis, and a history of cranial radiation therapy [13, 18, 19, 37] has revealed that astrocytes are a major cell type in the central nervous system that undergoes cellular senescence both in vitro and in vivo. Normal human astrocytes cultured in vitro became senescent following extensive cell divisions (replicative senescence) [13], as well as upon exposure to irradiation [18] or amyloid-β [19] (stress-induced senescence), with increased production of neurotoxic SASP cytokines and diminished expression of endogenous Δ133p53α [13, 18, 19]. Lentiviral expression of Δ133p53α protected these astrocytes from replicative or stress-induced senescence, accompanied by not only reduced levels of neurotoxic SASP cytokines but also enhanced production of neurotrophic growth factors [13, 18, 19]. These Δ133p53α-induced secretory changes in normal astrocytes contributed to improved neuronal survival in astrocyte-neuron co-culture experiments [13, 18, 19], warranting further investigation into the neuroprotective activities of Δ133p53α in vivo. Details of astrocyte-mediated neurotoxicity and neuroprotection, as well as the potential therapeutic effects of Δ133p53α in neurodegenerative diseases, have been summarized and discussed elsewhere [38].

## Δ133p53α-mediated protection of progeria patient-derived cells from premature senescence and accelerated DNA damage

Hutchinson-Gilford Progeria Syndrome (HGPS), a premature aging disorder affecting children, is caused by a splicing mutation in the *LMNA* gene, which generates an aberrant form of the lamin A protein called progerin [39, 40]. The progerin-induced abnormality in the nuclear envelope causes accumulated DNA damage, leading to hyperactivation of p53, premature induction of cellular senescence, and increased secretion of proinflammatory SASP cytokines [41–43]. Given that Δ133p53α functions to inhibit p53-mediated senescence, mitigate SASP, and preserve genome stability in normal human cells, as mentioned above, we investigated whether Δ133p53α can also protect against these progerin-induced changes. When Δ133p53α was lentivirally expressed in two strains of primary fibroblasts derived from HGPS patients, the cells underwent 12 or 20 additional population doublings, compared to control cells, before reaching replicative senescence [20]. These Δ133p53α-expressing, lifespan-extended cells showed reduced expression of the p53-inducible senescence genes p21^WAF1^ and miR-34a, reduced production of the SASP cytokines IL-6 and IL-8, and decreased accumulation of DNA double-strand breaks (DSB) [20]. An E2F1-mediated upregulation of a homologous recombination DNA repair factor RAD51 [44] was induced by Δ133p53 expression, likely contributing to enhanced repair of progerin-induced DSB [20]. These data suggest that Δ133p53α can mitigate the accelerated cellular aging phenotypes exhibited in HGPS patient-derived cells. Notably, Δ133p53α-expressing HGPS cells maintained high levels of progerin and still showed abnormal nuclear morphology [20], indicating that Δ133p53α acts on progerin-induced downstream events, rather than directly on progerin itself. We thus propose that a Δ133p53α-based therapeutic strategy could cooperate or synergize with currently tested progerin-targeting therapies for HGPS [45, 46].

## Lung epithelial cell senescence as a potential target for Δ133p53α enhancement

**Fig. 2:**
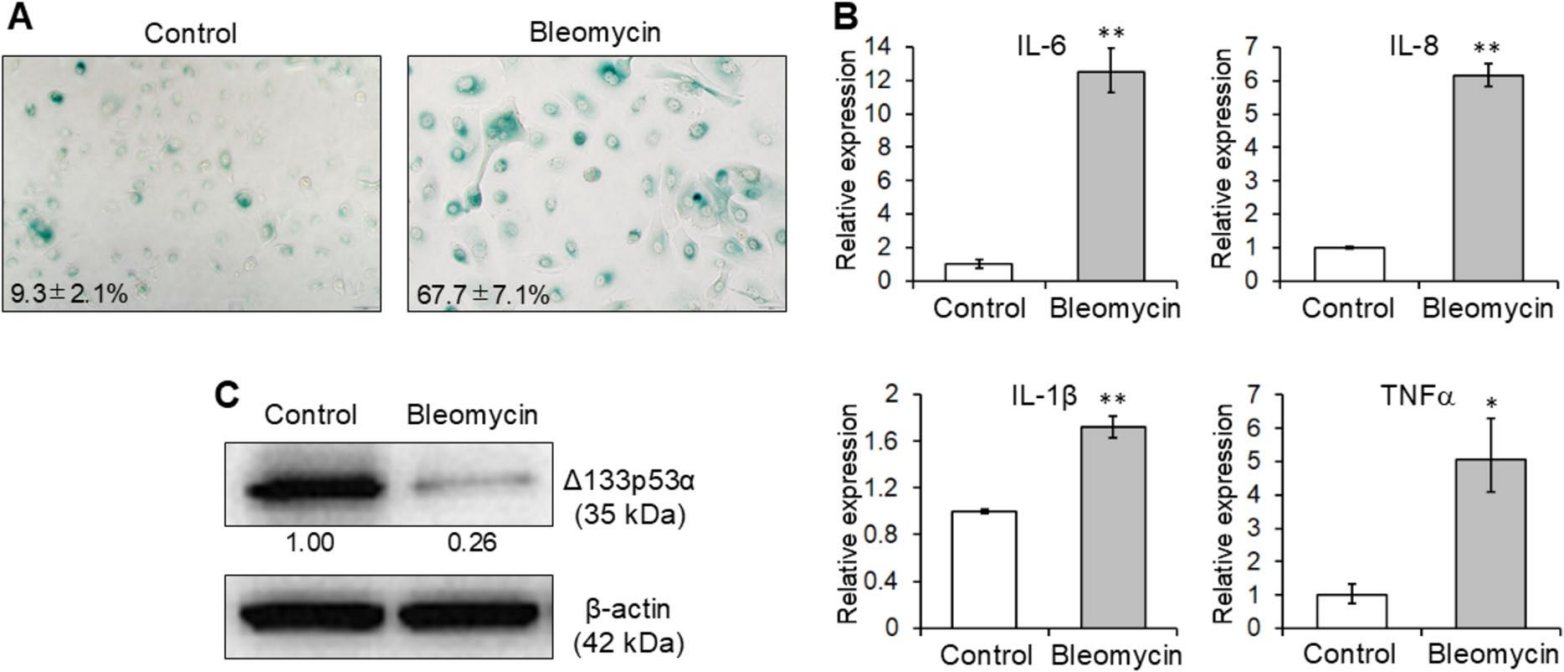
Reduced expression of Δ133p53α in senescent lung epithelial cells. Primary human bronchial epithelial cells (ScienCell Research Laboratories, Carlsbad, CA; catalog no. 3210) were exposed to bleomycin sulfate (Sigma-Aldrich, Rockville, MD; catalog no. B5507) at 1 mU/ml for 20 h, along with phosphate-buffered saline with no drug (control), followed by 5-day incubation before the following assays. **A** Senescence-associated β-galactosidase staining. Quantitative data (mean ± SD) were obtained from biological triplicates, in which at least 100 cells were observed per replicate and positively stained cells were recorded. **B** qRT-PCR assays of the SASP cytokines IL-6, IL-8, IL-1β, and TNFα. Expression data were normalized with GAPDH, and the relative values are shown as mean ± SD. The qRT-PCR assay reagents were purchased from Thermo Fisher Scientific (Carlsbad, CA): Assay ID Hs00985639_m1, Hs00174103_m1, Hs01555410_m1, Hs00174128_m1, and Hs02758991_g1 for IL-6, IL-8, IL-1β, TNFα, and GAPDH, respectively. *p < 0.05, **p < 0.01. **C** Western blot analysis of Δ133p53α protein expression. Δ133p53α was detected using its specific antibody [16]. β-actin (detected using catalog no. AM4302, Thermo Fisher Scientific) was a loading control. The relative expression values were from the quantitative image analysis using Image Lab software (Bio-Rad Laboratories, Hercules, CA)

Idiopathic pulmonary fibrosis (IPF) is the most common type of interstitial pneumonia, which primarily affects the elderly, progresses steadily over months to years, shows poor prognosis with a median survival of less than 4 years, and therefore urgently requires improvements in current therapies and the development of new therapeutic approaches [47]. The cellular etiology of IPF has long been linked to cellular senescence in both fibroblastic and epithelial cells [48]. A growing body of recent evidence, however, suggests that cellular senescence with SASP in lung epithelial cells (particularly alveolar type II cells) acts as an initiating event to trigger the pathological changes in IPF, such as interstitial inflammation and fibroblastic proliferation [49, 50]. p53 reportedly plays an essential role in lung epithelial cell senescence in IPF [50–52]. While various endogenous stressors (e.g., aging-associated oxidative damage and inflammation) and environmental risk factors (e.g., occupational exposures and cigarette smoking) can induce p53-mediated senescence in lung epithelial cells [47, 53], bleomycin is widely used in both human cell culture models of lung epithelial senescence [54] and mouse models of IPF [55, 56]. To examine a possible change in Δ133p53α expression during lung epithelial senescence, primary human bronchial epithelial cells [57, 58] were treated with bleomycin (Fig. 2). Consistent with previous reports [49, 54, 59], significant induction of cellular senescence (as indicated by SA-β-gal staining) (Fig. 2A) and increased expression of SASP cytokines such as IL-6, IL-8, IL-1β, and TNFα (Fig. 2B) were observed. Of particular importance, endogenous expression of Δ133p53α was markedly reduced in these bleomycin-treated, senescent bronchial epithelial cells (Fig. 2C), as previously observed in other types of senescent human cells [13, 15–20]. These results suggest that lung epithelial cell senescence may be a viable target for enhancing Δ133p53α, a natural inhibitor of p53-mediated senescence, with potential therapeutic implications for IPF.

## Δ133p53α transgenic mice to investigate in vivo therapeutic effects

**Fig. 3:**
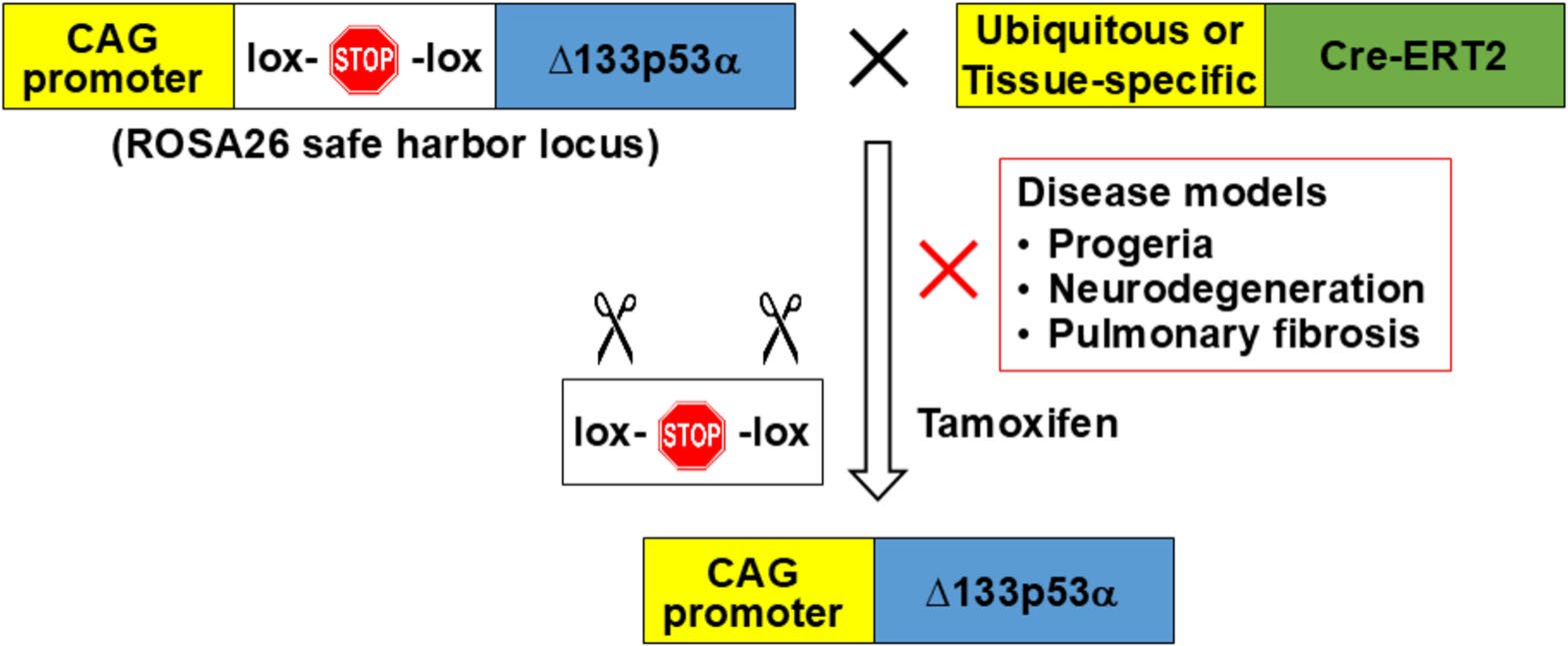
A mouse model for investigating therapeutic effects of Δ133p53α in vivo. The transgene consisting of the CAG promoter, the loxP-Stop-loxP cassette, and the Δ133p53α coding sequence has been knocked-in at the safe harbor ROSA26 locus. Crossbreeding with a strain ubiquitously expressing the tamoxifen-regulated Cre recombinase (Cre-ERT2), followed by tamoxifen administration, has induced the transgenic Δ133p53α expression in all the organs examined. Various tissue-specific Cre-ERT2 strains are also to be crossbred with this Δ133p53α transgenic strain, depending on the organ and cell type primarily affected in various disease models. Based on our data from cell culture studies, progeria syndromes such as HGPS, Alzheimer’s and other neurodegenerative diseases, and IPF are of particular interest

Further data supporting the beneficial effects of Δ133p53α in vivo are required to advance toward potential therapeutic applications. Although Δ133p53α is a human/primate-specific p53 isoform [13], lentiviral vector-driven Δ133p53α physically interacted with mouse full-length p53 protein and inhibited p53-mediated senescence in mouse embryonic fibroblasts (L. Yamada et al., unpublished data). Based on this functional compatibility of human Δ133p53α in mice, we have generated a transgenic mouse strain capable of inducible Δ133p53α expression (Fig. 3; L. Yamada et al., unpublished data). This mouse strain will be available through the Mutant Mouse Resource & Research Centers (ID 75789: https://www.mmrrc.org/). Inducible expression of Δ133p53α was confirmed by crossbreeding with a strain ubiquitously expressing Cre-ERT2 recombinase [60] (strain #007001, Jackson Laboratory), followed by intraperitoneal injection of tamoxifen. These crossbred mice, along with control mice, are currently being examined to assess the in vivo effects of Δ133p53α on physiological aging. As of 22 months of age after the systemic induction of Δ133p53α at 8 weeks of age, these mice show no increase in spontaneous tumorigenesis (L. Yamada et al., unpublished data), in contrast to highly tumorigenic p53-knockout mice, all of which developed spontaneous tumors by 6 months of age [24].

The Δ133p53α × Cre-ERT2 crossbred mice have been further crossbred with a HGPS mouse model [61] to investigate whether systemic expression of Δ133p53α can protect against the accelerated aging phenotypes characteristic of progeria (L. Yamada et al., unpublished data). The Δ133p53α transgenic strain can also be crossed with various tissue-specific Cre-ERT2 strains, depending on the diseases of interest (Fig. 3). For studies on IPF, an alveolar type II cell-specific Cre-ERT2 strain is available [62], and a widely used protocol for bleomycin-induced pulmonary fibrosis in mice is well established [55, 56]. For studies on neurodegenerative diseases, an astrocyte-specific Cre-ERT2 strain is available [63], and various mouse disease models and neurodegeneration-inducing protocols can be employed [64–68]. We propose that other disease models involving p53-mediated senescence are also worth investigating for the potential therapeutic effects of Δ133p53α. Our Δ133p53α transgenic mice thus represent a valuable tool for basic and translational research on Δ133p53α nationwide and worldwide.

## Future perspectives

Senescent cells are thought to consist of highly heterogeneous cell populations, likely ranging from reversible to irreversible states [69–71]. Senolytic drugs, which eliminate senescent cells, have emerged as a therapeutic approach for aging-associated diseases, including neurodegenerative diseases [72] and IPF [73, 74] in early-phase clinical trials, as well as HGPS in mouse models [75, 76]. We hypothesize that, while these senolytic drugs preferentially target irreversibly senescent cells, Δ133p53α primarily prevents cells in a reversible state from progressing to irreversible senescence and restores their proliferative and functional capacities (i.e., stem-like features in CD8^+^ T cells, neuroprotective activity in astrocytes, and enhanced DNA repair in HGPS cells). Cooperative or synergistic effects of senolytic and Δ133p53α-enhancing approaches are to be investigated in the above-mentioned mouse models.

Although our current data suggest that Δ133p53α is non-mutagenic and non-oncogenic, the potential risks of unintended consequences arising from Δ133p53α expression should be carefully considered and closely monitored in future therapeutic applications. Given the physiological roles of cellular senescence in tumor suppression, embryonic development, wound healing, and tissue regeneration [1–7], the Δ133p53α-mediated inhibition of cellular senescence requires strategic planning for appropriate timing and duration, organ-specific targeting, and the selection of suitable patients. In the aforementioned approaches combining senolytic drugs and Δ133p53α expression, SASP inhibition by Δ133p53α could potentially interfere with SASP-mediated immunosurveillance and clearance of senescent cells [77, 78]. This may lead to a tissue microenvironment where senescent cells persist, making it more susceptible to aging-associated changes and tumorigenesis [2, 79]. The cell culture and mouse models discussed in this review article can be utilized to investigate whether such adverse interaction could occur with the combination of senolytic drugs and Δ133p53α expression.

Toward clinical applications, administration and delivery methods for Δ133p53α-enhancing therapeutics need to be considered and developed. Recent advances in lipid nanoparticle (LNP)-based methods may enable organ-specific delivery of synthetic Δ133p53α mRNA [80, 81]. A newly developed LNP delivery system with anti-inflammatory properties [82] may be utilized to carry Δ133p53α-expressing vectors. Lastly, we have performed a high-throughput screening of small-molecule libraries and identified two prototype drugs that increase the endogenous expression of Δ133p53α in normal human astrocytes [83].

## Data Availability

For any inquiries, please contact I.H. at horikawi@mail.nih.gov.
